# The relationship between perceived social support and insufficient milk supply in primiparous mother

**DOI:** 10.1590/1980-220X-REEUSP-2025-0035en

**Published:** 2025-07-28

**Authors:** İrem Bengisu Öcal, Tuba Güner Emül

**Affiliations:** 1 Mersin University, Mersin, Turkey.; 2 Mersin University, Faculty Nursing, Mersin, Turkey.

**Keywords:** Parity, Breastfeeding, Social Support, Milk Ejection, Paridade, Aleitamento Materno, Apoio Social, Ejeção Láctea

## Abstract

**Objective:**

The objective of this study is to determine the relationship between perceived social support (MSPSS) and perceived insufficient milk supply (PIM) in primiparous mothers.

**Methods:**

This descriptive and cross-sectional study was conducted on 149 primiparous mothers who visited the gynecology and obstetrics outpatient clinics of a public hospital in Adana province in Turkey. Data were collected using the Descriptive Information Form, the Multidimensional Scale of Perceived Social Support (MSPSS), and the Perception of Insufficient Milk Questionnaire (PIM).

**Results:**

The mean scores obtained from the MSPSS (56.49 ± 20.19) and the PIM (27.46 ± 14.51) were above the moderate levels. A positive, moderate, and statistically significant correlation was observed between the total MSPSS and PIM scores (p < 0.001).

**Conclusion:**

Consequently, indicate a correlation between an increase in the level of MSPSS in primiparous mothers and a corresponding decrease in their PIM levels.

## RESUMO

**Objetivo:**

O objetivo deste estudo é determinar a relação entre o suporte social percebido (MSPSS) e a percepção de produção insuficiente de leite (PIM) em mães primíparas.

**Métodos:**

Este estudo descritivo e transversal foi realizado com 149 mães primíparas que visitaram as clínicas ambulatoriais de ginecologia e obstetrícia de um hospital público na província de Adana, na Turquia. Os dados foram coletados por meio do Formulário de Informações Descritivas, da Escala Multidimensional de Suporte Social Percebido (MSPSS) e do Questionário de Percepção de Leite Insuficiente (PIM).

**Resultados:**

As médias das pontuações obtidas na MSPSS (56,49 ± 20,19) e no PIM (27,46 ± 14,51) foram superiores aos níveis moderados. Observou-se uma correlação positiva, moderada e estatisticamente significativa entre os escores totais da MSPSS e do PIM (p < 0,001).

**Conclusão:**

Consequentemente, indica-se uma correlação entre o aumento no nível de suporte social percebido pelas mães primíparas e uma diminuição correspondente nos níveis de percepção de leite insuficiente.

## INTRODUCTION

Breast milk is a readily available and cost-effective food source that provides the essential nutrients required for infant growth and development^(1)^. It is well documented that breast­feeding represents the optimal method for delivering breast milk to the infant^(1)^. Notwithstanding the recommendations of the World Health Organization (WHO) and the United Nations International Children’s Emergency Fund (UNICEF) that infants should be exclusively breastfed for the first six months after birth, the global exclusive breastfeeding rate for infants under six months of age is only 48%^(2,3)^. According to the Turkish Demographic and Health Survey (TDHS) 2018 data, only 41% of infants under six months of age in Turkey are exclusively breastfed^(4)^. Despite the global prevalence of breast­feeding, numerous challenges impede mothers from initiating or sustaining this practice^(1)^.

The process of breastfeeding is influenced by a multitude of factors, including sociodemographic and physical characteristics of the woman, psychosocial variables, and social and environmental factors^(5)^. Among these factors, perceived social support (PSS) has been demonstrated to exert a considerable influence on breastfeeding practices^(5,6)^. A review of the literature revealed that mothers with high levels of PSS experienced fewer difficulties with breastfeeding^(7,8)^. It can therefore be concluded that the MSPSS levels of breastfeeding mothers are a significant factor in the continuation of breastfeeding.

Another significant factor influencing the duration of breastfeeding is the mother’s perception that her milk is inadequate to meet her infant’s nutritional needs and that her infant is not fully nourished^(9)^. Maternal concerns regarding the quantity and quality of milk are defined as perceived insufficient milk supply (PIM)^(9)^. A review of the literature indicates that the primary factors contributing to PIM include infants who frequently cry, suck, or reject breastfeeding, as well as mothers’ perceptions that their breasts have not been fully emptied^(9,10)^. These factors are misleading symptoms that cause the mothers’ PIM and may result in the interruption or termination of breastfeeding^(5,10)^. Moreover, the number of deliveries has been identified as a contributing factor to the PIM^(10,11)^. A number of studies have indicated that breastfeeding experience and knowledge have a positive impact on the PIM^(11,12)^. A review of the literature indicates that primiparous mothers tend to perceive their milk as less adequate than multiparous mothers^(5,13)^.

It is imperative to assess the perceptions of primiparous mothers regarding insufficient milk supply and social support, as these factors significantly influence their ability to maintain and improve their breastfeeding practices. A review of the existing literature revealed a paucity of research on this subject. This study, therefore, aimed to enhance awareness of the significance of MSPSS and PIM in the postpartum period, to direct attention to these concepts among healthcare professionals, to elucidate the relationship between the MSPSS and PIM among primiparous mothers, and to contribute to the existing literature on the subject. The objective of this study is to determine the relationship between MSPSS and insufficient milk supply in primiparous mothers.

The research questions included the following:

- What is the level of MSPSS in primiparous mothers?
- What is the level of perceived insufficient milk supply in primiparous mothers?
- Is there a relationship between the levels of MSPSS and insufficient milk supply in primiparous mothers?

## Methods

### Desiign of Study

The study had a descriptive and cross-sectional design. According to the Turkish Demographic and Health Survey (TDHS) 2018 data, only 41% of infants under six months of age are breastfed. This study was conducted in a province located in the southern region of Turkey. In this province, individuals with different socio-economic characteristics who migrate from many regions of the country live. The study population consisted of primiparous mothers who visited the gynecology and obstetrics outpatient clinics of a public hospital in Adana a province.

### Populatiion and Local

The study sample was selected using the purposive sampling method. The requisite sample size was determined through the use of the software program www.e-picos.com ^(14)^. A biostatistician was consulted for the calculation of the requisite sample size. A minimum sample size of 90 primiparous mothers was calculated to be required for a power of 0.80, a type I error of 0.05, and a correlation of 0.30 between the total scores obtained from the Multidimensional Scale of Perceived Social Support (MSPSS) and the Perception of Insufficient Milk (PIM) Questionnaire. The study included voluntary primiparous mothers who had undergone vaginal delivery, initiated breastfeeding at least once, and whose infants were at most six months of age. In consideration of the potential for withdrawals or data loss, the study was completed with the participation of 149 primiparous mothers.

### Measurement

The data were collected using the Descriptive Information Form, the Multidimensional Scale of Perceived Social Support (MSPSS), and the Perception of Insufficient Milk (PIM) Questionnaire.

### Descriptive Information Form

The form was developed by the researchers in accordance with the relevant literature^(6,7,8,9)^. The form includes questions pertaining to the mother’s sociodemographic characteristics, such as age, employment status, income level, educational attainment, type of family, and health insurance coverage, as well as breastfeeding-related characteristics, such as the type of first nutrition after birth, plans to exclusively breastfeed, assistance during the first breastfeeding, and training on breastfeeding. The form was developed with the input of three faculty members with expertise in the relevant fields.

### Multidimensional Scale of Perceived Social Support (MSPSS)

**Figure 1:**
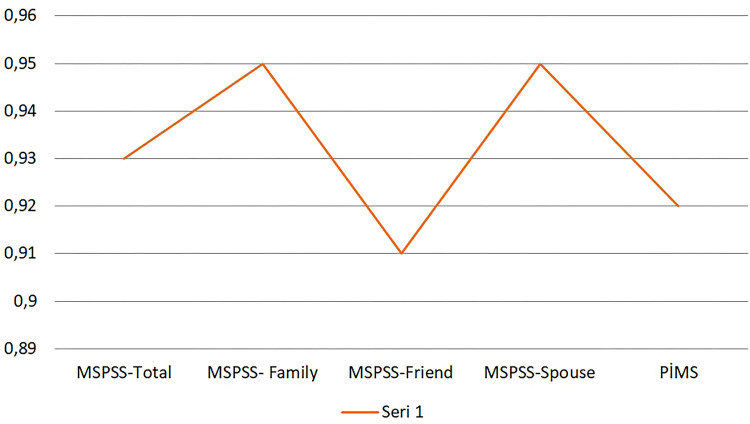
The Cronbach’s alpha coefficients of the MSPSS and PIM.

The scale was first developed in 1988 and adapted to Turkish in 1995^(15,16)^. The scale is composed of 12 items, which are organized into three subscales: family, friends, and a significant other. The items are scored on a 7-point Likert scale, with responses ranging from 1 (very strongly disagree) to 7 (very strongly agree). The range of possible scores is from 12 to 84, with higher scores indicating higher levels of PSS. The Cronbach’s alpha coefficient for the Turkish version of the scale and its subscales ranged from 0.80 to 0.95. In our study, the Cronbach’s alpha coefficient for the MSPSS and its subscales ranged from 0.91 to 0.95 (Figure 1).

### Perception of Insufficient Milk (PIM) Questionnaire

The PIM questionnaire was first developed in 2001 and adapted to Turkish in 2014^(8,17)^. The questionnaire comprises six items. The initial question, which queries the respondent’s perception of their ability to produce sufficient milk to meet their infant’s nutritional needs, is answered dichotomously (i.e., yes or no). The remaining five questions are responded to on a 10-point Likert scale, with scores ranging from 0 (strongly disagree) to 10 (strongly agree). The range of possible scores is from 0 to 50, with higher scores indicating a lower level of perceived insufficient milk supply. The Cronbach’s alpha coefficients of the Turkish version of the questionnaire and in our study were 0.82 and 0.92, respectively (Figure 1).

### Data Collection

The data were collected between April and June 2024 via face-to-face interviews. After being informed about the aim of the study, the participants were asked to complete all questions on the data collection tool, which took approximately 15 minutes.

### Data Analysis

SPSS version 21.0 was used for data analysis. Number, percentage, mean, standard deviation, and minimum and maximum values were used to present descriptive data. The normality and homogeneity of the data were tested using the Shapiro-Wilk and Levene tests. A Student’s t-test was employed to compare two independent groups, while the one-way ANOVA test was used to compare three or more independent groups. In instances where a significant difference was identified, the Tukey test was utilized as a post hoc analysis. Pearson’s correlation test was used to analyze the relationship between continuous variables. Statistical significance was set at p < 0.05.

### Ethical Considerations

Before the data collection tools were applied, written ethics committee approval was obtained from Toros University Clinical Research Ethics Committee with the Board Decision dated 26/01/2024 and numbered 2024/31 and written institutional permission was obtained from the Ministry of Health Adana Provincial Health Directorate with the decision numbered E-11289099-050.04-240896094 and dated 05/04/2024. After the necessary explanations were made to the mothers included in the study about the purpose of the research, the method of application and the results planned to be obtained, their consent was obtained.

## RESULTS

Table 1 presents the sociodemographic characteristics of the participants. The data indicate that 56.3% of the participants had obtained a university degree, 59.1% were employed, 91.9% had health insurance coverage, 83.9% belonged to a nuclear family, and 65.8% had an income that equaled their expenses.

**Table 1:** Sociodemographic characteristics – Adana, Türkiye, 2024.

Variables	$\bar{X}\pm \text{SD}$	Min–Max
Age	25.4 ± 4.1	17–36
	n	%
Educational attainment		
Primary & secondary school	27	18.2
High school	38	25.5
University and above	84	56.3
Employment status		
Employed	61	40.9
Unemployed	88	59.1
Type of family		
Nuclear	125	83.9
Extended	24	16.1
Has a health insurance		
Yes	137	91.9
No	12	8.1
Income level		
Less than expenses	40	26.8
Equal to expenses	98	65.8
More than expenses	11	7.4

**Figure 2:**
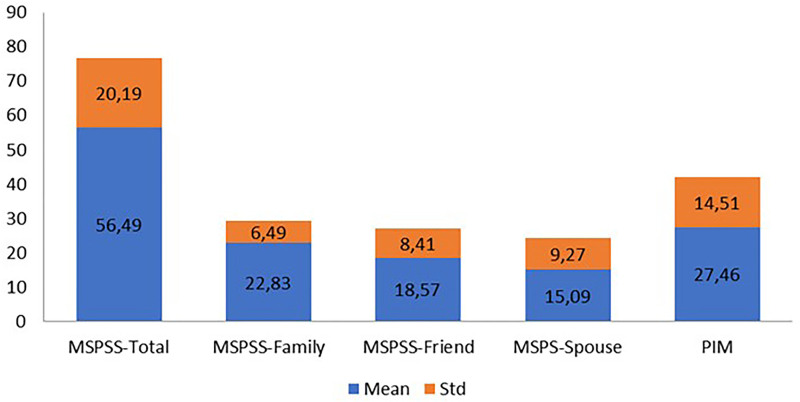
The scores obtained from the MSPSS and the PIM.

Table 2 presents the characteristics regarding breastfeeding. The majority of the mothers (82.6%) fed their infants using colostrum, more than half (57.7%) planned to exclusively breastfeed the infant up to six months of age, and 88.6% received assistance during the first breastfeeding attempt, with midwives and nurses constituting 74.2% of this assistance. Figure 2 illustrates the scores obtained from the MSPSS and the PIM. The mean scores obtained from the MSPSS and its subscales of family, friends and significant other were 56.49 ± 20.19, 22.83 ± 6.49, 18.57 ± 8.41 and 15.09 ± 9.27, respectively. The mean score obtained from the PIM was 27.46 ± 14.51.

**Table 2:** Characteristics regarding breastfeeding – Adana, Türkiye, 2024.

Variables	n	%
First nutrition after birth		
Colostrum	123	82.6
Formula	16	10.7
Other	10	6.7
Plans to exclusively breastfeed the infant up to		
6^th^ month	86	57.7
1 year old	48	32.2
2 years old	15	10.1
Received assistance during the first breastfeeding		
Yes	132	88.6
No	17	11.4
Received assistance from (n = 132)*		
Midwife/Nurse	98	74.2
Friends/Relatives	34	25.8
Received training on breastfeeding		
Yes	111	74.5
No	38	25.5
Received the training from (n = 111)**		
Midwife/Nurse	100	90.1
Physician	11	9.9

Table 3 presents the relationship between the scores obtained from the MSPSS and the PIM. A positive, moderate, and statistically significant correlation was observed between the total MSPSS and PIM scores (p < 0.001). Additionally, a positive, weak, and statistically significant correlation was observed between the scores obtained from the PIM and the family, friends, and significant other subscales of the MSPSS (p < 0.001).

**Table 3:** Relationship between the MSPSS and the PIM Questionnaire Scores – Adana, Türkiye, 2024.

	PIMQ		
	r		p
MSPSS	0.40		< 0.001
Family	0.35		<0.001
Friends	0.35		< 0.001
A significant other	0.30		< 0.001

r: Pearson’s correlation coefficient.

## DISCUSSION

The postpartum period is a period during which the mother requires the greatest amount of social support, given the addition of new roles and responsibilities, as well as the psychological and physiological changes that occur^(18)^. Insufficient social support during this period may result in a decline in maternal mental health, challenging maternal adaptation to the motherhood process, and disruption of breastfeeding^(18,19)^. The support that primiparous mothers receive from their social environment, particularly from their spouses, has been demonstrated to positively influence the process of adaptation to motherhood and infant care, as well as the success of breastfeeding^(9,20)^. A new mother must maintain both mental and physical health in order to sustain her own well-being and provide optimal care for her infant^(19)^. It is therefore crucial for the mother to perceive that she is receiving social support^(8,19)^. A substantial body of research indicates that social support during the postpartum period has a pronounced impact on mothers’ breastfeeding behaviors^(19,20)^.

The study revealed that the MSPSS levels of primiparous mothers were above the moderate levels (56.49 ± 20.19), with the greatest amount of support being received from family (22.83 ± 6.49) (Figure 2). Other studies have similarly reported elevated levels of MSPSS among mothers, with the majority of support derived from family^(21,22,23)^. In contrast with the aforementioned findings, studies conducted in China and Vietnam have indicated that mothers’ MSPSS levels were insufficient, with the least support being provided by family^(24,25)^. The elevated level of familial support observed in the present study and in previous investigations conducted on the Turkish population may be attributed to the cultural values of the Turkish people, which place a high importance on solidarity and mutual support within the family. Furthermore, as the participants in the present study were primiparous mothers, they received support and assistance from elder family members, which may have contributed to an increase in the level of MSPSS. It is therefore recommended that nurses plan breastfeeding trainings and counseling services with a holistic approach and include the family members of primiparous mothers in these trainings.

The study revealed that the lowest level of social support was provided by a significant other (15.09 ± 9.27), which encompasses individuals other than friends and family members, such as partners, fiancées, spouses, relatives, neighbors, or health professionals^(14)^. As all participants in this study were married, it is unsurprising that they obtained the lowest score from the significant other subscale of the MSPSS. These findings differ found that 90% of the mothers received social support from their partners, which could be considered an example of significant other support^(26)^. This discrepancy may be explained by differing cultural definitions of the concept of “a significant other.”

The PIM presents a significant challenge to the practice of exclusive breastfeeding for the first six months postpartum, with adverse implications for breastfeeding^(23)^. In the event that the mother is unable to resolve the issues she encounters during breastfeeding, she may resort to complementary feeding due to the belief that she is unable to feed her infant. This creates a vicious cycle for breastfeeding^(24)^. Such circumstances may result in adverse health outcomes for both the mother and the infant in the short and long term^(6)^. Furthermore, unnecessary complementary feeding and the preference for formula over breast milk may result in adverse health outcomes in the short term and later in life^(27,28)^.

The mean PIM score of the present study was above the moderate levels (27.46 ± 14.51), indicating that the participants perceived their milk supply to be adequate. These findings indicate the success of breastfeeding policies in Türkiye. A review of the extant literature reveals that the PIM is affected by the number of deliveries and previous experience of breastfeeding, with primiparous mothers obtaining lower scores from the PIM^(11,12)^. A review of the literature on primiparous mothers reveals a tendency for them to report lower levels of PIM than observed in our study^(12)^. This discrepancy may be attributed to differences in the educational level of the participants in our study, as well as their level of knowledge about breast milk and breastfeeding. On the other hand, a comparison of primiparous and multiparous mothers indicates that the latter group reported a lower level of PIM^(28)^. This is an anticipated outcome that reflects the breastfeeding experience of multiparous mothers. Moreover, the studies in question were conducted at disparate time points, spanning a period of 0–24 months postpartum, whereas the present study included mothers with infants of at most six months of age. Therefore, it can be posited that the participants of this study may exhibit a greater degree of concern regarding milk adequacy, whereas the mothers in the aforementioned studies may demonstrate a lesser degree of concern due to their experience and maternal knowledge regarding breastfeeding, which may have increased over the subsequent months following birth. Other studies conducted in Australia, the USA, and Ethiopia reported PIM in 44%, 66%, and 83% of the participants, respectively^(10,29,30)^. These findings indicate that there is a discrepancy between mothers’ perception of adequate milk and the desired level, that the PIM varies across cultures, and that it is a pervasive issue.

The findings of our study demonstrated that an increase in the perceived level of social support among primiparous mothers was associated with a corresponding increase in the perception of adequate milk supply (p < 0.001). Moreover, the social support received from family, friends, and a significant other was found to have a positive effect on the perception of adequate milk supply (p < 0.001). The lack of experience that primiparous mothers have in matters such as infant care and breastfeeding, in addition to their need to adapt to the role of motherhood in the postpartum period, increases the necessity for support from their spouses, family members, and the social environment^(10,16)^. It is thus anticipated that the MSPSS received from family, friends, and a significant other will have a positive effect on the mothers’ perception of adequate milk supply. Mobilizing social support systems in the postpartum period will have a beneficial effect on the breastfeeding process. A review of the existing literature reveals no studies that have examined the relationship between MSPSS and the PIM. The findings of our study underscore the correlation between the perceived level of social support and the perception of insufficient milk among primiparous mothers, as well as the significance of this relationship in the context of breastfeeding. Concurrently, the pivotal role of nurses in providing education and counseling, which can facilitate the prevention of inadequate milk perception, is further highlighted.

### Limitations

The study was conducted at a single health center, which may limit the generalizability of the findings to a broader population.

## CONCLUSION

In conclusion, in our study, it was determined that the social support perceived by primiparous mothers decreased the perception that their milk was insufficient. This situation suggests that while developing training and counselling services for primiparous mothers, social support is important in the breastfeeding process and making plans for this will contribute to a more enjoyable and longer breastfeeding process for mothers who experience breastfeeding for the first time.

## Data Availability

The data that support the findings of this study are available from the corresponding author upon reasonable request.
