# Supplementary material for: The relationship between perceived social support and insufficient milk supply in primiparous mother
Source: Rev Esc Enferm USP. 2025 Jul 28;59:e20250035. doi: 10.1590/1980-220X-REEUSP-2025-0035en (PMC12313323; doi:10.1590/1980-220X-REEUSP-2025-0035en)
Supplement: Translation

# Translation: La relación entre el apoyo social percibido y el suministro insuficiente de leche en madres primíparas

**DOI:** 10.1590/1980-220X-REEUSP-2025-0035es

**Authors:** İrem Bengisu Öcal, Tuba Güner Emül

**Affiliations:** 1 Mersin University, Mersin, Turkey.; 2 Mersin University, Faculty Nursing, Mersin, Turkey.

**Keywords:** Paridad, Lactancia Materna, Apoyo Social, Eyección Láctea

## Abstract

**Objetivo:**

El objetivo de este estudio es determinar la relación entre el apoyo social percibido (MSPSS) y la percepción de suministro insuficiente de leche (PIM) en madres primíparas.

**Métodos:**

Este estudio descriptivo y transversal se realizó con 149 madres primíparas que acudieron a las consultas externas de ginecología y obstetricia de un hospital público en la provincia de Adana, Turquía. Los datos se recolectaron utilizando el Formulario de Información Descriptiva, la Escala Multidimensional de Apoyo Social Percibido (MSPSS) y el Cuestionario de Percepción de Suministro Insuficiente de Leche (PIM).

**Resultados:**

Las puntuaciones medias obtenidas en la MSPSS (56,49 ± 20,19) y en la PIM (27,46 ± 14,51) fueron superiores a los niveles moderados. Se observó una correlación positiva, moderada y estadísticamente significativa entre las puntuaciones totales de la MSPSS y del PIM (p < 0,001).

**Conclusión:**

En consecuencia, se indica una correlación entre un aumento en el nivel de apoyo social percibido en madres primíparas y una disminución correspondiente en sus niveles de percepción de suministro insuficiente de leche.

## INTRODUCCIÓN

La leche materna es una fuente de alimentación fácilmente disponible y asequible que aporta los nutrientes esenciales necesarios para el crecimiento y el desarrollo del lactante^(1)^. Está bien documentado que la lactancia materna representa el método óptimo para suministrar leche materna al lactante^(1)^. A pesar de que la Organización Mundial de la Salud (OMS) y el Fondo Internacional de Emergencia de las Naciones Unidas para la Infancia (UNICEF) recomiendan que los lactantes reciban lactancia materna exclusiva durante los seis primeros meses de vida, la tasa mundial de lactancia materna exclusiva en menores de seis meses es sólo del 48%^(2,3)^. Según los datos de la Encuesta Demográfica y de Salud de Turquía (TDHS por su sigla en inglés) de 2018, solo el 41% de los lactantes menores de seis meses en Turquía se alimentan exclusivamente con leche materna^(4)^. A pesar de la prevalencia mundial de la lactancia materna, numerosos retos impiden a las madres iniciar o mantener esta práctica^(1)^.

El proceso de la lactancia materna está influenciado por multitud de factores, incluyendo características sociodemográficas y físicas de la mujer, variables psicosociales y factores sociales y ambientales^(5)^. Entre estos factores, se ha demostrado que el apoyo social percibido (APS) ejerce una influencia considerable en las prácticas de lactancia^(5,6)^. Una revisión de la literatura reveló que las madres con altos niveles de apoyo social percibido (MSPSS por su sigla en inglés) experimentaron menos dificultades con la lactancia^(7,8)^. Por lo tanto, se puede concluir que los niveles de MSPSS de las madres lactantes son un factor significativo en la continuidad de la lactancia materna.

Otro factor importante que influye en la duración de la lactancia materna es la percepción de la madre de que su leche es inadecuada para satisfacer las necesidades nutricionales de su hijo y de que éste no está plenamente nutrido^(9)^. La preocupación materna por la cantidad y la calidad de la leche se define como percepción de insuficiencia de leche (PIM por su sigla en inglés)^(9)^. Una revisión de la literatura indica que los principales factores que contribuyen al PIM incluyen a los lactantes que lloran, succionan o rechazan la lactancia con frecuencia, así como la percepción de las madres de que sus pechos no se han vaciado completamente^(9,10)^. Estos factores son síntomas engañosos que provocan el PIM de las madres y pueden dar lugar a la interrupción o finalización de la lactancia^(5,10)^. Además, el número de partos se ha identificado como un factor que contribuye al PIM^(10,11)^. Varios estudios han indicado que la experiencia y el conocimiento de la lactancia tienen un impacto positivo en el PIM^(11,12)^. Una revisión de la literatura indica que las madres primíparas tienden a percibir su leche como menos adecuada que las multíparas^(5,13)^.

Es imperativo evaluar las percepciones de las madres primíparas sobre la insuficiencia de leche y el apoyo social, ya que estos factores influyen significativamente en su capacidad para mantener y mejorar sus prácticas de lactancia. Una revisión de la literatura existente reveló la escasez de investigaciones sobre este tema. Este estudio, por lo tanto, pretendía aumentar la concienciación sobre la importancia del MSPSS y el PIM en el periodo posparto, dirigir la atención a estos conceptos entre los profesionales sanitarios, dilucidar la relación entre el MSPSS y el PIM entre las madres primíparas, y contribuir a la literatura existente sobre el tema. El objetivo de este estudio es determinar la relación entre el MSPSS e insuficiencia de leche en madres primíparas.

Las preguntas de la investigación eran las siguientes

- ¿Cuál es el nivel de MSPSS en las madres primíparas?
- ¿Cuál es el nivel de insuficiencia de leche percibida en las madres primíparas?
- ¿Existe una relación entre los niveles de MSPSS y la producción insuficiente de leche en madres primíparas?

## MÉTODOS

### Diseño del Estudio

El estudio tuvo un diseño descriptivo y transversal. Según los datos de la Encuesta Demográfica y de Salud de Turquía (TDHS) de 2018, solo el 41% de los lactantes menores de seis meses son amamantados. Este estudio se llevó a cabo en una provincia situada en la región sur de Turquía. En esta provincia viven individuos con diferentes características socioeconómicas que emigran de muchas regiones del país. La población del estudio estaba formada por madres primíparas que acudían a las consultas externas de ginecología y obstetricia de un hospital público de la provincia Adana.

### Población y Local

La muestra del estudio se seleccionó mediante el método de muestreo intencional. El tamaño requerido de la muestra se determinó mediante el programa informático www.e-picos.com^(14)^. Se consultó a un bioestadístico para calcular el tamaño requerido de la muestra. Se calculó que se necesitaba una muestra mínima de 90 madres primíparas para obtener una potencia de 0,80, un error de tipo I de 0,05 y una correlación de 0,30 entre las puntuaciones totales obtenidas de la Escala Multidimensional de Apoyo Social Percibido (Multidimensional Scale of Perceived Social Support - MSPSS) y el Cuestionario de Percepción de Leche Insuficiente (Perception of Insufficient Milk – PIM). El estudio incluyó a madres primíparas voluntarias que habían tenido un parto vaginal, habían iniciado la lactancia al menos una vez y cuyos bebés tenían como máximo seis meses de edad. Teniendo en cuenta la posibilidad de que se produjeran abandonos o pérdidas de datos, el estudio se completó con la participación de 149 madres primíparas.

### Medición

Los datos se recogieron utilizando el Formulario de Información Descriptiva, la Escala Multidimensional de Apoyo Social Percibido (MSPSS) y el Cuestionario de Percepción de Insuficiencia de Leche (PIM).

### Formulario de Información Descriptiva

El formulario fue desarrollado por los investigadores de acuerdo con la literatura relevante^(6,7,8,9)^. El formulario incluye preguntas relativas a las características sociodemográficas de la madre, como la edad, la situación laboral, el nivel de ingresos, el nivel educativo, el tipo de familia y la cobertura del seguro médico, así como características relacionadas con la lactancia, como el tipo de primera alimentación tras el parto, los planes de lactancia exclusiva, la asistencia durante la primera lactancia y la formación sobre lactancia. El formulario se elaboró con las aportaciones de tres profesores expertos en los campos correspondientes.

### Escala Multidimensional de Apoyo Social Percibido (MSPSS)

La escala se desarrolló por primera vez en 1988 y se adaptó al turco en 1995^(15,16)^. La escala se compone de 12 ítems, organizados en tres subescalas: familia, amigos y pareja. Los ítems se puntúan en una escala Likert de 7 puntos, con respuestas que van de 1 (muy en desacuerdo) a 7 (muy de acuerdo). El rango de puntuaciones posibles va de 12 a 84, y las puntuaciones más altas indican niveles más elevados de PSS. El coeficiente alfa de Cronbach para la versión turca de la escala y sus subescalas osciló entre 0,80 y 0,95. En nuestro estudio, el coeficiente alfa de Cronbach para la MSPSS y sus subescalas osciló entre 0,91 y 0,95 (Figura 1).

**Figura 1:** Coeficientes alfa de Cronbach del MSPSS y del PIM.

### Cuestionario Sobre la Percepción de la Insuficiencia de Leche (PIM)

El cuestionario PIM se desarrolló por primera vez en 2001 y se adaptó al turco en 2014^(8,17)^. El cuestionario consta de seis preguntas. La pregunta inicial, que cuestiona la percepción del encuestado sobre su capacidad para producir suficiente leche para satisfacer las necesidades nutricionales de su bebé, se responde de forma dicotómica (es decir, sí o no). Las cinco preguntas restantes se responden en una escala de Likert de 10 puntos, con puntuaciones que van de 0 (totalmente en desacuerdo) a 10 (totalmente de acuerdo). El rango de puntuaciones posibles va de 0 a 50, indicando las puntuaciones más altas un nivel más bajo de percepción de insuficiencia de suministro de leche. Los coeficientes alfa de Cronbach de la versión turca del cuestionario y en nuestro estudio fueron de 0,82 y 0,92, respectivamente (Figura 1).

### Reco Lección de Datos

Los datos se recogieron entre abril y junio de 2024 mediante entrevistas personales. Tras ser informados sobre el objetivo del estudio, se pidió a los participantes que completaran todas las preguntas de la herramienta de recogida de datos, lo que llevó aproximadamente 15 minutos.

### Análisis de Datos

Para el análisis de los datos se utilizó el programa SPSS versión 21.0. Para presentar los datos descriptivos se utilizaron el número, el porcentaje, la media, la desviación típica y los valores mínimo y máximo. La normalidad y homogeneidad de los datos se comprobaron mediante las pruebas de Shapiro-Wilk y Levene. Para comparar dos grupos independientes se empleó la prueba t de Student, mientras que para comparar tres o más grupos independientes se utilizó la prueba ANOVA unidireccional. En los casos en que se identificó una diferencia significativa, se utilizó la prueba de Tukey como análisis post hoc. Para analizar la relación entre variables continuas se utilizó la prueba de correlación de Pearson. La significación estadística se fijó en p < 0,05.

### Consideraciones Éticas

Antes de aplicar los instrumentos de recogida de datos, se obtuvo la aprobación por escrito del Comité Ético de Investigación Clínica de la Universidad Toros, con la Decisión de la Junta fechada el 26/01/2024 y numerada 2024/31, y se obtuvo el permiso institucional por escrito de la Dirección Provincial de Sanidad del Ministerio de Sanidad Adana, con la decisión numerada E-11289099-050.04-240896094 y fechada el 05/04/2024. Tras las explicaciones necesarias a las madres incluidas en el estudio sobre la finalidad de la investigación, el método de aplicación y los resultados que se pretendía obtener, se obtuvo su consentimiento.

## RESULTADOS

La Tabla 1 presenta las características sociodemográficas de los participantes. Los datos indican que el 56,3% de los participantes habían obtenido un título universitario, el 59,1% estaban empleados, el 91,9% tenían cobertura de seguro médico, el 83,9% pertenecían a una familia nuclear y el 65,8% tenían unos ingresos que igualaban sus gastos.

**Tabla 1:** Características sociodemográficas Características socio­demográficas – Adana, Turquía, 2024.

Variables	$\bar{X}\pm \text{SD}$	Min–Max
Edad	25.4 ± 4.1	17–36
	n	%
Nivel de estudios		
Enseñanza primaria y secundaria	27	18.2
Bachillerato	38	25.5
Universitarios y superiores	84	56.3
Situación laboral		
Empleado	61	40.9
Desempleado	88	59.1
Tipo de familia		
Nuclear	125	83.9
Ampliado	24	16.1
Tiene un seguro de enfermedad		
Sí	137	91.9
No	12	8.1
Nivel de ingresos		
Menos que gastos	40	26.8
Igual a gastos	98	65.8
Más que gastos	11	7.4

La Tabla 2 presenta las características relativas a la lactancia materna. La mayoría de las madres (82,6%) alimentó a sus hijos con calostro, más de la mitad (57,7%) tenía previsto amamantar exclusivamente al niño hasta los seis meses de edad y el 88,6% recibió asistencia durante el primer intento de amamantamiento, de la que el 74,2% correspondió a matronas y enfermeras. La Figura 2 ilustra las puntuaciones obtenidas con el MSPSS y el PIM. Las puntuaciones medias obtenidas del MSPSS y sus subescalas de familia, amigos y pareja fueron 56,49 ± 20,19, 22,83 ± 6,49, 18,57 ± 8,41 y 15,09 ± 9,27, respectivamente. La puntuación media obtenida del PIM fue de 27,46 ± 14,51.

**Tabla 2:** Características relativas a la lactancia materna – Adana, Türkiye, 2024.

Variables	n	%
Primera alimentación tras el parto		
Calostro	123	82.6
Fórmula	16	10.7
Otros	10	6.7
Planes para amamantar exclusivamente al bebé hasta		
6^o^ mes	86	57.7
1 año	48	32.2
2 años	15	10.1
Recibió asistencia durante el primer lactancia materna		
Sí	132	88.6
No	17	11.4
Recibió ayuda de (n = 132)*		
Matrona/Enfermera	98	74.2
Amigos/familiares	34	25.8
Recibió formación sobre lactancia materna		
Sí	111	74.5
No	38	25.5
Recibió la formación de (n = 111)**		
Partera/Enfermera	100	90.1
Médico	11	9.9

**Figura 2:** Escores de MSPSS y PIM.

La Tabla 3 presenta la relación entre las puntuaciones obtenidas en el MSPSS y el PIM. Se observó una correlación positiva, moderada y estadísticamente significativa entre las puntuaciones totales del MSPSS y el PIM (p < 0,001). Además, se observó una correlación positiva, débil y estadísticamente significativa entre las puntuaciones obtenidas en el PIM y las subescalas de familia, amigos y otros significativos del MSPSS (p < 0,001).

**Tabla 3:** Relación entre las puntuaciones del MSPSS y las del cuestionario PIM (PIMQ) – Adana, Turquía, 2024.

	PIMQ		
	r		p
MSPSS	0.40		< 0.001
Familia	0.35		< 0.001
Amigos	0.35		< 0.001
Pareja (“otro/a significativa”)	0.30		< 0.001

r: Coeficiente de correlación de Pearson.

## DISCUSIÓN

El puerperio es un periodo durante el cual la madre requiere la mayor cantidad de apoyo social, dada la incorporación de nuevas funciones y responsabilidades, así como los cambios psicológicos y fisiológicos que se producen^(18)^. Un apoyo social insuficiente durante este periodo puede provocar un deterioro de la salud mental materna, dificultar la adaptación de la madre al proceso de maternidad e interrumpir la lactancia^(18,19)^. Se ha demostrado que el apoyo que las madres primíparas reciben de su entorno social, en particular de sus cónyuges, influye positivamente en el proceso de adaptación a la maternidad y al cuidado del bebé, así como en el éxito de la lactancia^(9,20)^. Una madre primeriza debe mantener una buena salud mental y física para mantener su propio bienestar y proporcionar unos cuidados óptimos a su hijo^(19)^. Por lo tanto, es crucial que la madre perciba que recibe apoyo social^(8,19)^. Numerosas investigaciones indican que el apoyo social durante el puerperio tiene un gran impacto en el comportamiento de las madres en relación con la lactancia^(19,20)^.

El estudio reveló que los niveles de MSPSS de las madres primíparas estaban por encima de los niveles moderados (56,49 ± 20,19), y que el mayor apoyo lo recibían de la familia (22,83 ± 6,49) (Figura 2). Otros estudios han informado de forma similar de niveles elevados de MSPSS entre las madres, con la mayor parte del apoyo derivado de la familia^(21,22,23)^. En contraste con los hallazgos mencionados anteriormente de, estudios realizados en China y Vietnam han indicado que los niveles de MSPSS de las madres eran insuficientes, y que el menor apoyo lo proporcionaba la familia^(24,25)^. El elevado nivel de apoyo familiar observado en el presente estudio y en investigaciones anteriores realizadas en la población turca puede atribuirse a los valores culturales del pueblo turco, que concede una gran importancia a la solidaridad y al apoyo mutuo dentro de la familia. Además, como las participantes en el presente estudio eran madres primíparas, recibían apoyo y ayuda de familiares mayores, lo que puede haber contribuido a aumentar el nivel de MSPSS. Por lo tanto, se recomienda que las enfermeras planifiquen los cursos de formación y los servicios de asesoramiento sobre lactancia materna con un enfoque holístico e incluyan a los familiares de las madres primíparas en estos cursos.

El estudio reveló que el nivel más bajo de apoyo social lo proporcionaba una persona significativa (15,09 ± 9,27), que engloba a personas distintas de amigos y familiares, como parejas, novios, cónyuges, parientes, vecinos o profesionales de la salud^(14)^. Dado que todos los participantes en este estudio estaban casados, no es de extrañar que obtuvieran la puntuación más baja en la subescala del otro significativo del MSPSS. Estos resultados difieren en que el 90% de las madres recibían apoyo social de sus parejas, lo que podría considerarse un ejemplo de apoyo del otro significativo^(26)^. Esta discrepancia puede explicarse por las diferentes definiciones culturales del concepto de “otro significativo”.

El PIM supone un reto importante para la práctica de la lactancia materna exclusiva durante los primeros seis meses posparto, con implicaciones adversas para la lactancia^(23)^. En caso de que la madre no pueda resolver los problemas que se le plantean durante la lactancia, puede recurrir a la alimentación complementaria debido a la creencia de que es incapaz de alimentar a su hijo. Esto crea un círculo vicioso para la lactancia^(24)^. Estas circunstancias pueden tener consecuencias negativas para la salud de la madre y el lactante a corto y largo plazo^(6)^. Además, la alimentación complementaria innecesaria y la preferencia por la leche artificial frente a la materna pueden tener consecuencias negativas para la salud a corto plazo y en etapas posteriores de la vida^(27,28)^.

La puntuación media del PIM del presente estudio estaba por encima de los niveles moderados (27,46 ± 14,51), lo que indica que las participantes percibían que su suministro de leche era adecuado. Estos resultados indican el éxito de las políticas de lactancia materna en Turquía. Una revisión de la literatura existente revela que el PIM se ve afectado por el número de partos y la experiencia previa de lactancia, obteniendo las madres primíparas puntuaciones más bajas en el PIM^(11,12)^. Una revisión de la literatura sobre madres primíparas revela una tendencia a que éstas informen de niveles más bajos de PIM que los observados en nuestro estudio^(12)^. Esta discrepancia puede atribuirse a las diferencias en el nivel educativo de las participantes en nuestro estudio, así como a su nivel de conocimientos sobre la leche materna y la lactancia. Por otro lado, la comparación entre madres primíparas y multíparas indica que este último grupo informó de un nivel más bajo de PIM^(28)^. Se trata de un resultado esperado que refleja la experiencia de lactancia de las madres multíparas. Además, los estudios en cuestión se llevaron a cabo en momentos dispares, abarcando un periodo de 0 a 24 meses posparto, mientras que el presente estudio incluyó a madres con bebés de seis meses de edad como máximo. Por lo tanto, se puede postular que las participantes de este estudio pueden mostrar un mayor grado de preocupación con respecto a la adecuación de la leche, mientras que las madres de los estudios mencionados pueden demostrar un menor grado de preocupación debido a su experiencia y conocimiento materno con respecto a la lactancia materna, que puede haber aumentado durante los meses posteriores al parto. Otros estudios realizados en Australia, EE.UU. y Etiopía informaron de PIM en el 44%, 66% y 83% de las participantes, respectivamente^(10,29,30)^. Estos resultados indican que existe una discrepancia entre la percepción que tienen las madres de la leche adecuada y el nivel deseado, que el PIM varía según las culturas y que es un problema generalizado.

Los resultados de nuestro estudio demostraron que un aumento en el nivel percibido de apoyo social entre las madres primíparas se asoció con un aumento correspondiente en la percepción de un suministro adecuado de leche (p < 0,001). Además, se observó que el apoyo social recibido de la familia, los amigos y la pareja tenía un efecto positivo en la percepción de un suministro de leche adecuado (p < 0,001). La falta de experiencia que tienen las madres primíparas en temas como el cuidado del bebé y la lactancia materna, además de su necesidad de adaptarse al rol de la maternidad en el puerperio, aumenta la necesidad de apoyo por parte de sus cónyuges, familiares y el entorno social^(10,16)^. Por lo tanto, se prevé que el MSPSS recibido de la familia, los amigos y una persona significativa tendrá un efecto positivo en la percepción de las madres de un suministro de leche adecuado. La movilización de los sistemas de apoyo social en el periodo posparto tendrá un efecto beneficioso en el proceso de lactancia. Una revisión de la literatura existente no revela ningún estudio que haya examinado la relación entre el MSPSS y el PIM. Los resultados de nuestro estudio subrayan la correlación entre el nivel de apoyo social percibido y la percepción de insuficiencia de leche entre las madres primíparas, así como la importancia de esta relación en el contexto de la lactancia materna. Al mismo tiempo, se pone de relieve el papel fundamental que desempeñan las enfermeras en la educación y el asesoramiento, que pueden facilitar la prevención de la percepción de leche insuficiente.

### Limitaciones

El estudio se realizó en un único centro de salud, lo que puede limitar la generalización de los resultados a una población más amplia.

## CONCLUSIÓN

En conclusión, en nuestro estudio se determinó que el apoyo social percibido por las madres primíparas disminuyó la percepción de que su leche era insuficiente. Esta situación sugiere que mientras se desarrollan servicios de formación y asesoramiento para madres primíparas, el apoyo social es importante en el proceso de lactancia y hacer planes para ello contribuirá a un proceso de lactancia más agradable y prolongado para las madres que experimentan la lactancia por primera vez.

## Data Availability

Los datos que respaldan los hallazgos de este estudio están disponibles a través del autor de correspondencia previa solicitud razonable.
